# Structure Features and Anti-Gastric Ulcer Effects of Inulin-Type Fructan CP-A from the Roots of *Codonopsis pilosula* (Franch.) Nannf.

**DOI:** 10.3390/molecules22122258

**Published:** 2017-12-18

**Authors:** Jiankuan Li, Tao Wang, Zhichuan Zhu, Fengrong Yang, Lingya Cao, Jianping Gao

**Affiliations:** 1 School of Pharmaceutical Science, Shanxi Medical University, No. 56 Xinjian South Road, Taiyuan 030001, China; jiankuanli@sxmu.edu.cn (J.L.); wt142701@163.com (T.W.); zhuzhichuan1126@163.com (Z.Z.); yfr152@126.com (F.Y.); 2 School of Basic Medical Science, Shanxi Medical University, No. 56 Xinjian South Road, Taiyuan 030001, China; caolingyablue2008@163.com

**Keywords:** *Codonopsis pilosula* (Franch.) Nannf., inulin-type fructan, gastric ulcer, structure determination, carbohydrate

## Abstract

Radix Codonopsis has been used in traditional Chinese medicine for strengthening the immune system, improving poor gastrointestinal function, treating gastric ulcers and chronic gastritis and so on. In the present study, an inulin-type fructan CP-A was obtained from the roots of *Codonopsis pilosula* (Franch.) Nannf. and its structure was confirmed by MS and NMR as (2 → 1) linked-β-d-fructofuranose. The protective effects of CP-A against ethanol-induced acute gastric ulcer in rats were intensively investigated. A Lacy assay demonstrated that CP-A-treated group (50 mg/kg) showed the gastric damage level 1, which was similar to the positive control group, while the model group exhibited the gastric damage level 3. The Guth assay demonstrated that the mucosa ulcer index for CP-A groups at the doses of 50 mg/kg and 25 mg/kg significantly decreased compared with that in the model group (*p* < 0.05). Meanwhile, CP-A significantly increased the activities of SOD and GSH-Px, and decreased the contents of MDA and NO, and the activity of MPO in gastric tissue in a dose-dependent manner (*p* < 0.05). The present research reported for the first time that inulin-type fructan CP-A were likely the potential component in Radix Codonopsis for treatment of acute gastric ulcers.

## 1. Introduction

Radix Codonopsis is recorded in the Chinese Pharmacopoeia as the dried roots of *Codonopsis pilosula* (Franch.) Nannf., *Codonopsis pilosula* (Franch.) Nannf. var. modesta (Nannf.) L. T. Shen, and *Codonopsis tangshen Oliv.*, and has been used in traditional Chinese medicine for replenishing *qi* deficiency, strengthening the immune system, improving poor gastrointestinal function, treating gastric ulcers and chronic gastritis and so on [1]. Phytochemical studies indicate that polysaccharides, alkaloids, phenylpropanoids, triterpenes, and polyacetylenes are the main components in Radix Codonopsis [1]. Recently, more and more attention has been paid to the study of polysaccharides from Radix Codonopsis. Several such polysaccharides are obtained from Radix Codonopsis and some exhibit potential bioactivities [2,3,4,5,6,7]. 

Fructan is a general term used for any carbohydrate which contains one or more fructosyl-fructose links in its chemical structure. Inulin-type fructan is defined as a carbohydrate consisting mainly of β (2 → 1) fructosyl-fructose links. Owing to the β configuration of the anomeric carbon, inulin-type fructans are resistant to hydrolysis by human digestive enzymes in the gastrointestinal tract, so inulin-type fructans are often fermented to produce short-chain carboxylic acids such as acetate, butyrate and propionate other than fructose during their passage through the gastrointestinal tract [8,9]. Some inulin-type fructans used for prebiotics have been reported to exhibit diverse functions such as regulation of blood sugar and lipid, gastrointestinal health, anticancer, mineral metabolism and bone remodeling and so on [10]. Recent findings suggest that inulin-type fructans exhibit an effect on the human gut microbiota [11]. 

The present research reported an inulin-type fructan CP-A isolated from the dried roots of *Codonopsis pilosula* (Franch.) Nannf. and its anti-gastric ulcer effect for the first time, which demonstrated that inulin-type fructans were likely the potential components in Radix Codonopsis responsible for the activity against gastric ulcers. 

## 2. Results and Discussion

### 2.1. Structure Identification of CP-A

**Figure 1:**
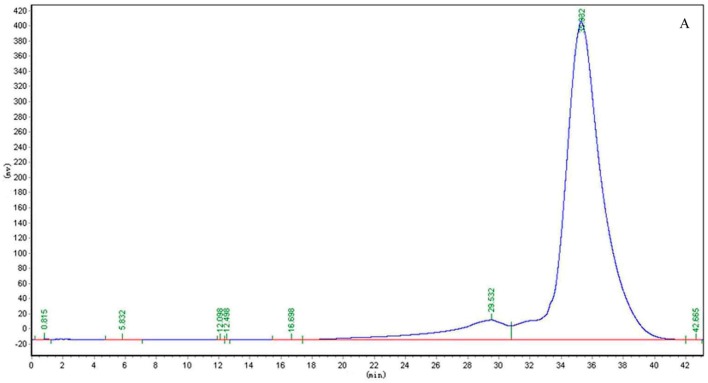

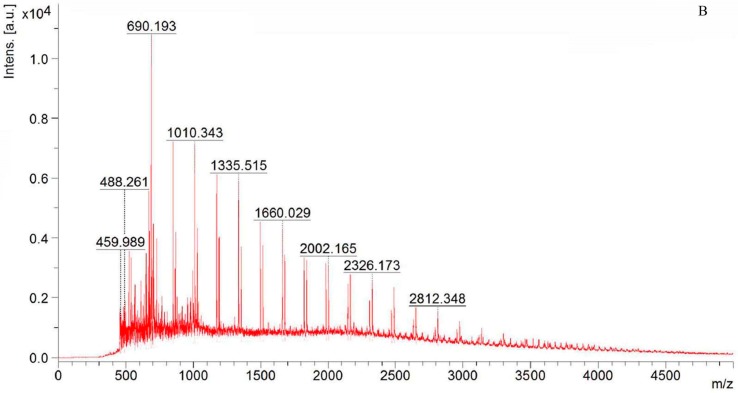
HPGPC curve (**A**) and MALDI-TOF mass spectrum (**B**) of CP-A.

**Figure 2:**
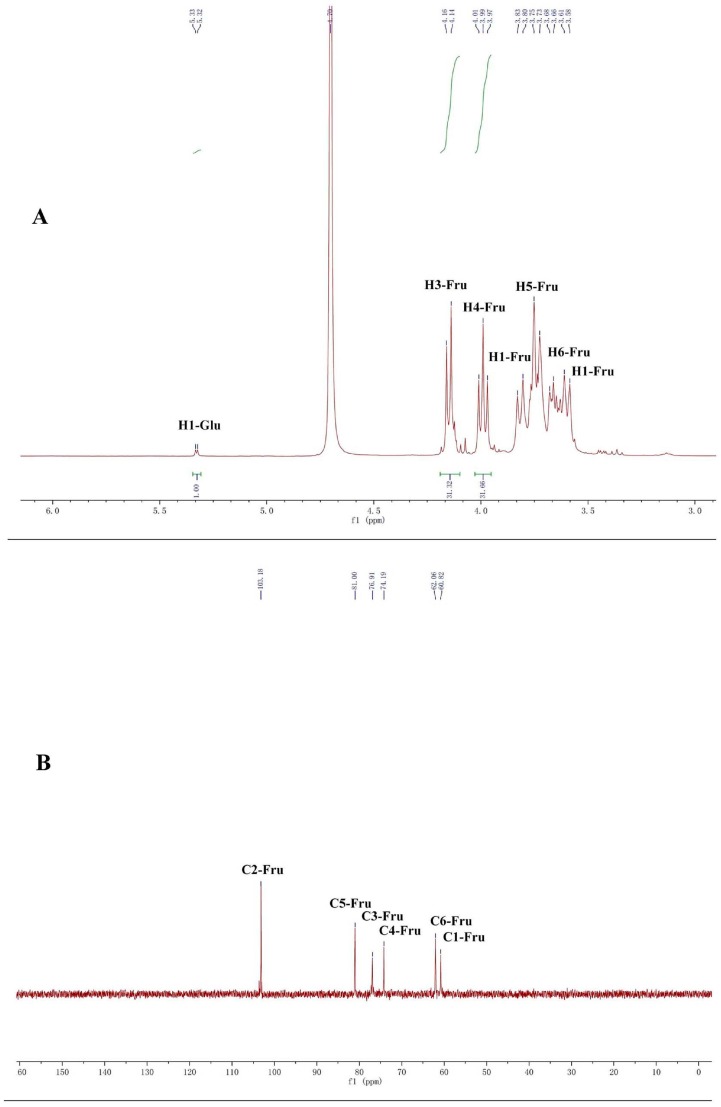

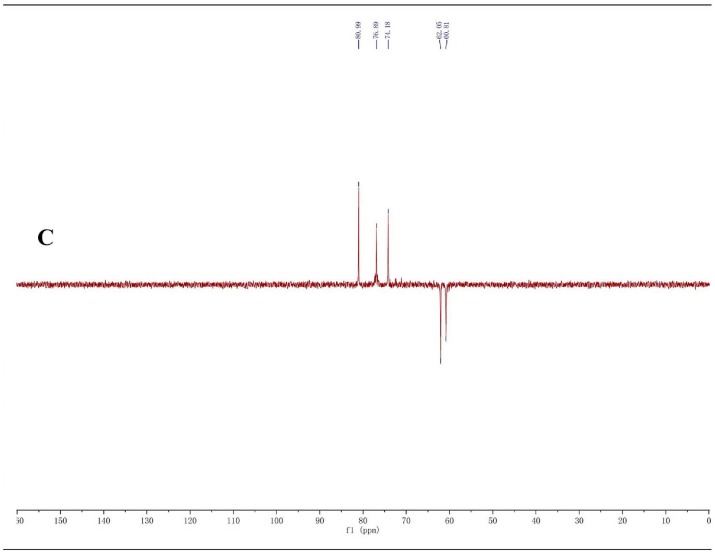
^1^H-NMR (**A**); ^13^C-NMR (**B**) and DEPT135 (**C**) spectra of CP-A.

**Figure 3:**
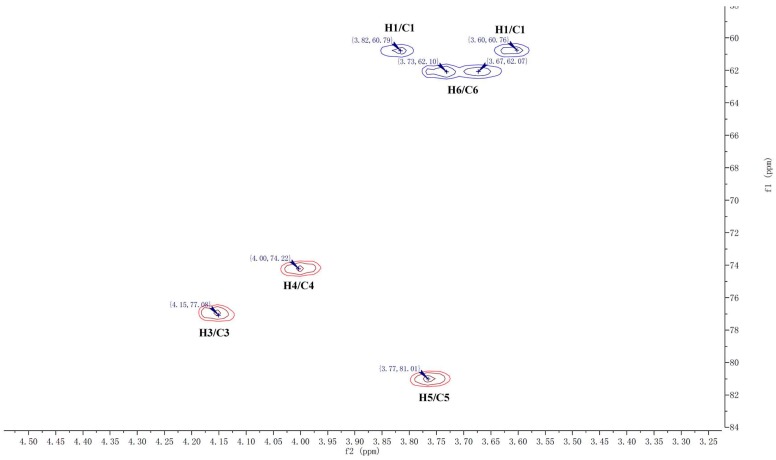
HSQC spectrum of CP-A.

**Figure 4:**
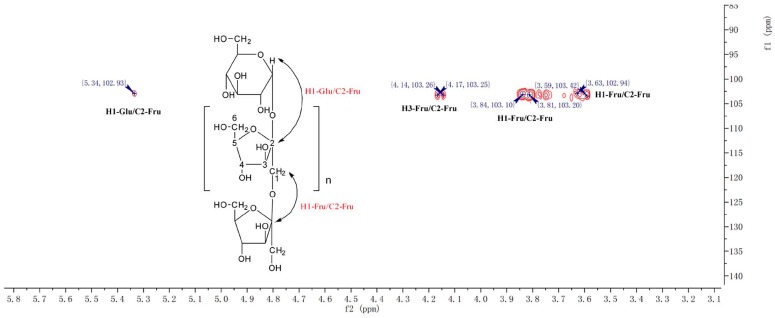
HMBC spectrum of CP-A.

CP-A exhibited a single and symmetrically sharp peak on HPGPC (Figure 1A) indicating its homogeneity. According to the retention time, its molecular weight was estimated to be 3.6 KDa. In the ^1^H-NMR spectrum of CP-A (Figure 2A), several signals from 3.50 ppm to 5.50 ppm occur, which were assigned as hydrogens on sugars, and the assignment of H signals is shown in Figure 1A. In the ^13^C-NMR spectrum (Figure 2B), six carbon signals occurred at 103.2, 81.2, 77.0, 74.3, 62.0 and 61.0 ppm. From the DEPT135 spectrum (Figure 2C), it was demonstrated that the carbon at 103.2 ppm was a quaternary carbon and the signals at 62.00 and 60.98 ppm were assigned to methylenes, while three other signals at 81.25, 77.06, 74.26 ppm were assigned to methines, which were consistent with the HSQC spectrum (Figure 3). The HMBC spectrum showed correlations between the C2-Fru/H1-Fru of fructosyl residues and indicated the presence of (2 → 1)-linked β-d-fructofuranosyl (Figure 4). In combination with the literature data [8,12,13,14], it was concluded that CP-A exhibited an inulin-type fructan chain structure (as shown in Figure 4), which was consistent with the results of a methylation assay and Smith degradation (data not shown). Meanwhile, as shown in Figure 2A, the integration ratio of H1-Glu to that of H3-Fru was 1:30, which indicated that the degree of polymerization (DP) of CP-A was 31 [8,12]. The MALDI-TOF mass spectrum (Figure 1B) was similar to that of an inulin-type fructan standard and there was a mass difference of 162 Da between two neighboring ions in the spectrum, which corresponds to fructose/fructose residues [12].

### 2.2. Effect of CP-A on Rat Gastric Mucosal Morphology

The gastric mucosa plays an important role in the physiological function of the stomach by acting as a gastric barrier protecting deeper located cells from injury caused by gastric secretory components including acid and pepsin. The gastric ulcer is a multifactorial disease which is mainly caused by stress, infection, administration of steroidal and non-steroidal anti-inflammatory drugs, tobacco smoking and alcohol intake [15]. Ethanol-induced gastric ulcer is a key experimental model widely used for assessment of potential anti-ulcer agents since ethanol has been regarded as a leading cause of gastric ulcer in humans and the gastric ulcer induced by ethanol is characterized pathologically by hemorrhage, edema, inflammatory response, and epithelial cell loss [16,17,18,19].

**Figure 5:**
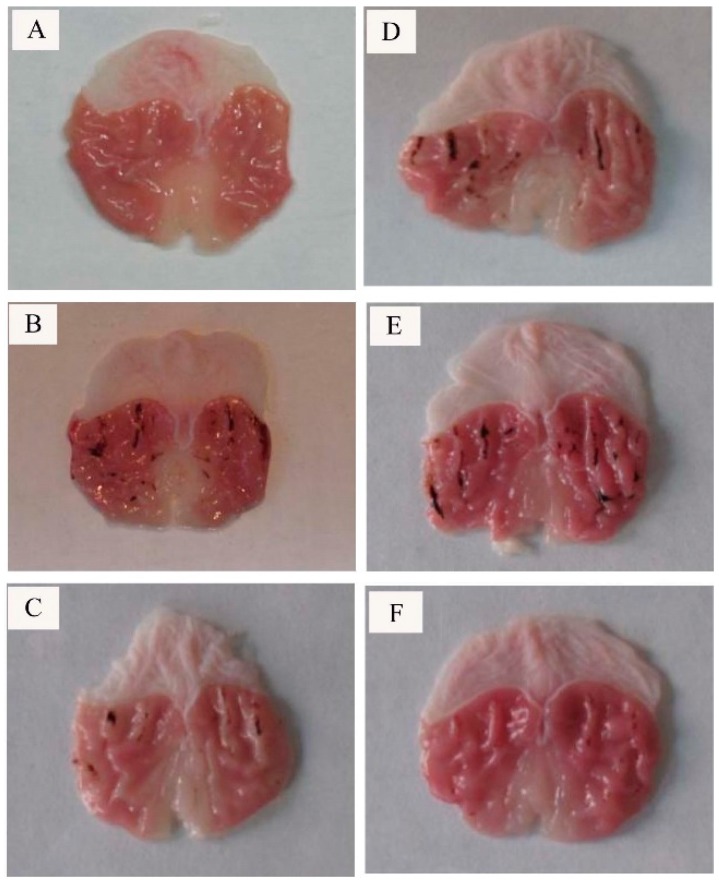
Morphological appearance of different treatments against ethanol-induced gastric lesions. (**A**) Normal; (**B**) Ethanol; (**C**) CP-A 50 mg/kg + Ethanol; (**D**) CP-A 25 mg/kg + Ethanol; (**E**) CP-A 12.5 mg/kg + Ethanol and (**F**) BPC + Ethanol.

In the present research, the rat gastric mucosal in normal group was smooth and no bleeding while the rat gastric mucosal in model group exhibited serious bleeding injury. Positive control group, high (50 mg/kg) and medium (25 mg/kg) dosage of CP-A group showed significantly inhibitory effects on bleeding injury of gastric mucosal in comparison with model group (Ethanol group) (Figure 5).

### 2.3. Effects of CP-A on Rat Gastric Mucosa Damage

**Figure 6:**
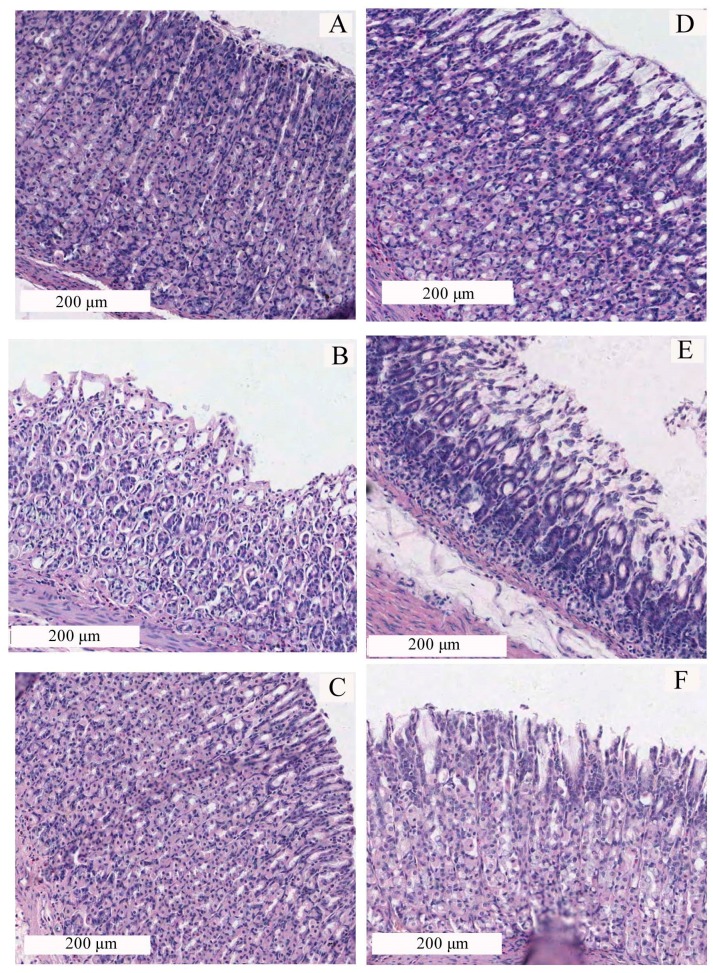
Histological sections of different treatments against ethanol-induced gastric lesions (Original magnification 200×). (**A**) Normal; (**B**) Ethanol; (**C**) CP-A 50 mg/kg + Ethanol; (**D**) CP-A 25 mg/kg + Ethanol; (**E**) CP-A 12.5 mg/kg + Ethanol and (**F**) BPC + Ethanol.

The Lacy assay is usually used for grading gastric mucosa damage [20]. According to the Lacy assay, rat gastric mucosa epithelium in the normal group was intact without abnormal epithelial necrosis or falling off and the glands were neat, which supported the assignment of damage degree as the damage level 0 for the normal group. Rat gastric mucosa epithelium in the model group exhibited obvious thinning, shedding and necrosis, and ulcer gastric epithelial cells disorderly arranged and loose, which was evaluated as damage level 3. Rat gastric mucosa damage for the positive control group was evaluated as level 1 due to its normal epithelium and some loosely arranged cells. Rat gastric mucosa damage for the high dosage (50 mg/kg) of CP-A group was assigned as the level 1 owing to its neat epithelium and some necrotic cells. However, rat gastric mucosa epithelium in the medium (25 mg/kg) and low dosage (12.5 mg/kg) of CP-A groups exhibited similar damage to the model group (Ethanol group) and was assigned as the damage level 3 (Figure 6). 

### 2.4. Effects of CP-A on Rat Gastric Mucosa Ulcer Index

**Figure 7:**
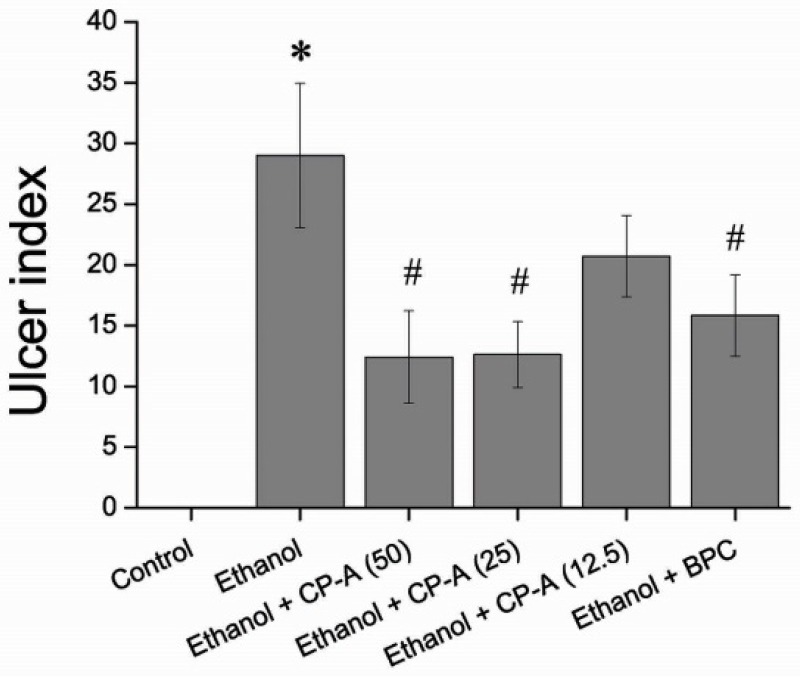
Effect of CP-A on ulcer index in ethanol-induce rat gastric mucosal. Data are presented as mean ± SD (*n* = 10). * *p* < 0.05 versus Normal group; ^#^ *p* < 0.05 versus Ethanol group.

As shown in Figure 7, the mucosa ulcer index for high (50 mg/kg) and medium dosage (25 mg/kg) of CP-A groups decreased significantly compared with the model group (Ethanol group, *p* < 0.05), which was similar to positive control. However, the ulcer index for the low dosage (12.5 mg/kg) of CP-A group showed no difference in comparison with the model group. 

### 2.5. Effects of CP-A on the Activities of MPO, SOD, GSH-Px and the Contents of MDA, NO in Gastric Tissue

Reactive oxygen species (ROS) such as superoxide anion, hydroxyl radical, and hydrogen peroxide are generated continuously during the process of respiration in aerobic organisms. Excessive generation of ROS exceeding the antioxidant capacity of organisms can cause oxidative stress. Superoxide dismutase (SOD) is one of the main antioxidant enzymes whose function is to scavenge superoxide anion by accelerating its conversion to H_2_O_2_ which is eliminated by glutathione peroxidase (GSH-Px) and catalase to water. Malondialdehyde (MDA), as a lipid peroxidation marker, is commonly used to evaluate the oxidative and antioxidant status in cells [21,22]. Oxidative stress has been considered a key step for ethanol-induced gastric injury [23]. In the present study, it was found that the levels of antioxidant enzymes (SOD and GSH-Px) were markedly reduced and the content of MDA was significantly elevated in the ethanol-induced model group compared to that in the normal control group. However, CP-A-pretreated group showed obvious inhibitory effects on ethanol-induced reduction of SOD, GSH-Px levels and increase of MDA content, which demonstrated that CP-A exhibited antioxidant potential in ethanol-induced gastric ulcer in rats. 

Nitric oxide (NO) plays a key role in maintain of the gastric mucosal integrity and physiological function by regulating acid level, gastric mucus secretion, and blood flow in gastric tissues [24]. When gastric damage happens, the content of NO in stomach tissue would decrease dramatically [25,26]. Our results showed that ethanol caused a significant drop of NO content, while CP-A exhibited significant inhibitory effect on decrease of NO content induced by ethanol in gastric tissues. MPO is estimated to be involved in the development of acute gastric mucosal lesions and a reduction of MPO activity is considered as a manifestation of the anti-inflammatory activity [27,28].

**Figure 8:**
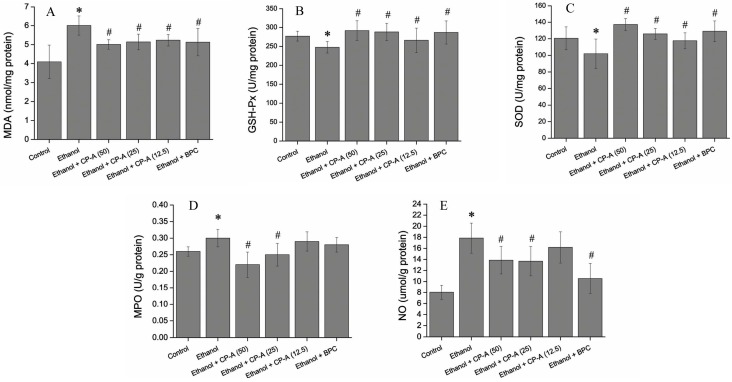
Effect of CP-A on MDA content (**A**); GSH-Px (**B**); SOD (**C**); MPO activity (**D**); and NO level (**E**) in ethanol-induce gastric mucosal. Data are presented as mean ± SD (*n* = 10). * *p* < 0.05 versus Normal group; ^#^ *p* < 0.05 versus Ethanol group.

As shown in Figure 8, the treatment of rats with 75% ethanol caused a significant decrease in SOD and GSH-Px activities (*p* < 0.05) along with a significant increase in the production of MDA and NO and the activity of MPO compared with control group (*p* < 0.05). However, the treatment of rats with CP-A (50, 25 and 12.5 mg/kg) considerably attenuated the decreased activities of SOD and GSH-Px (*p* < 0.05) while prominently inhibited the increased the production of MDA and NO and the activity of MPO (*p* < 0.05) induced by ethanol in a dose-dependent manner. These data suggested that CP-A exhibited antioxidative effects and protection against ethanol-induced gastric tissue damage in rats. 

## 3. Material and Methods

### 3.1. Materials and Chemicals

Roots of *Codonopsis pilosula* (Franch.) Nannf. were collected from LinChuan County in Shanxi Province, China, and identified by Professor Jianping Gao. A voucher specimen (20150705) was deposited at the School of Pharmaceutical Science, Shanxi Medical University. Dextran 70, 180, 2500, 4600, 7100, 10,000 were obtained from National Institute for the Control of Pharmaceutical and Biological Products (Beijing, China). Assay kits for MPO, SOD, GSH-Px, MDA and NO were purchased from Nanjing Jiancheng Bioengineering Institute (Nanjing, China).

### 3.2. Extraction and Purification of CP-A

The air-dried root of *Codonopsis pilosula* (20 kg) was crushed and extracted three times with distilled water (20:1, *v*/*w*) at 85 °C for 2.5 h. The extract was concentrated in vacuum and was precipitated with 95% ethanol at a final concentration of 50% for 12 h at 4 °C and obtained crud polysaccharide after centrifugation at 5000 rpm for 5 min. The crud polysaccharide was washed with anhydrous ethanol, acetone and ether, successively. The washed crud polysaccharide was dissolved in water and separated by Prep-scale TFT Ultrafiltration apparatus (Millipore Corporation, Billerica, MA, USA) through 3000 grade membrane (Millipore Corporation) to obtain CP-A. Protein was removed from CP-A using the Sevag reagent (chloroform/butanol, *v*/*v* = 4:1). 

### 3.3. Homogeneity and Molecular Weight Determination of CP-A

The homogeneity and molecular weight of CP-A were determined by high performance gel permeation chromatography (HPGPC) performed with a LC-10AT HPLC system (SHIMADZU, Kytot, Japan) fitted with a Tskgel G4000 PWXL column (300 mm × 8 mm) and a Shodex RI-20H refractive index detector. The mobile phase was ultrapure water and the flow rate was 0.3 mL/min at 35 °C. The molecular weight of CP-A was estimated by reference to a calibration curve obtained from a set of Dextran standards of known molecular weight (Dextran 70, 180, 2500, 4600, 7100, 10,000).

### 3.4. NMR and TOF-MS Analysis of CP-A

Dried CP-A (10 mg) was dissolved in DMSO-*d*_6_ in 5-mm NMR tube and measured for ^1^H-NMR, ^13^C-NMR, DEPT, HSQC and HMBC on an AV-400 spectrometer (Bruker, Rheinstetten, Germany). The degree of polymerization (DP) of CP-A was calculated according to the mean ratio of the protons signals integral (H3-Fru and H4-Fru) by the integrals of the anomeric hydrogen glucose signal (H1-Glc) in the ^1^H-NMR spectrum. The TOF-MS was conducted using an Ultraflex MALDI-TOF/TOF spectrometer (Bruker Daltonics, Billerica, MA, USA). The spectra were obtained in positive-mode.

### 3.5. Anti-Gastric Ulcer Effect of CP-A in Ethanol-Induced Acute Gastric Ulcer Rats

Sprague Dawley rats (weighing 180 g–220 g) were provided by Experimental Animal Center of Shanxi Medical University. Animal experiments were carried out under principles in good laboratory animal care and approved by ethical committee for Laboratory Animals Care and Use of Shanxi Medical University (2017001). Sixty rats (30 males and 30 females) were acclimatized for 1 week prior to use. All rats were randomized into six groups consisting of ten rats per group. Normal and model control groups received normal saline at a dose of 5 mL/kg by gavage. Positive control group received bismuth potassium citrate (BPC) (Zhuhai Livzon Pharmaceutical Factory of LIVZON group, Zhuhai, China) at a dose of 100 mg/kg by gavage. Experimental CP-A groups were given by gavage at doses of 12.5, 25, 50 mg/kg, representative of low, medium, and high dosage, respectively. The rats were administered once per day for seven days and then fasted for 24 h and had free access to water. Three hours after the last administration, acute gastric ulcer was induced in rats of groups by gavage administration of 1 mL 75% ethanol per rat. One hour later, all rats were anesthetized by peritoneal injection of pentobarbital sodium at dose of 45 mg/kg and then sacrificed. Gastric tissues were immediately collected for gastric ulcer index determination by the Guth assay [29]. The other half of tissue fixed in 10% formaldehyde solution for haematoxylin eosin (HE) staining and the other half of tissue for the detection of MPO, SOD, GSH-Px activities and MDA, NO contents according to the corresponding kit manuals.

### 3.6. Statistic

The data were expressed as mean ± SD and Student’s *t*-test was used for comparisons between two groups. Statistical significance was defined as a *p*-value less than 0.05. 

## 4. Conclusions

The present study provided insights into the potential of CP-A, an inulin-type fructan isolated from roots of *Codonopsis pilosula* (Franch.) Nannf., to inhibit ethanol-induced ulcers in rats. The results indicated that the CP-A-treated group (50 mg/kg) showed level 1 gastric damage, which was similar to the positive control group, while the model group exhibited level 3 gastric damage according to the Lacy assay. A Guth assay demonstrated that the mucosa ulcer index for CP-A groups at the dose of 50 mg/kg and 25 mg/kg decreased significantly compared with that in model group (*p* < 0.05). Meanwhile, CP-A increased significantly the activities of SOD and GSH-Px, and decreased the contents of MDA and NO and the MPO activity of gastric tissue in a dose-dependent manner in ethanol-induced rats. (*p* < 0.05).

To our knowledge, it is the first report that inulin-type fructan from roots of *Codonopsis pilosula* (Franch.) Nannf exhibits antiulcer action. The present study demonstrated that the antiulcer activity of CP-A was likely associated with the modulation of antioxidant and anti-inflammatory effects, which demonstrated that inulin-type fructan was likely the active potential components in Radix Codonopsis for the treatment of acute gastric ulcers. The mechanisms for the antiulcer activity of CP-A need to be thoroughly studied in subsequent researches.

## Abbreviations

The following abbreviations are used in this manuscript:

BPC	bismuth potassium citrate
DEPT	distortionless enhancement by polarization transfer
DMSO	dimethyl sulphoxide
DP	degree of polymerization
GSH-Px	glutathione peroxidase
HMBC	heteronuclear multiple bond correlation
HPGPC	high performance gel permeation chromatography
HSQC	heteronuclear singular quantum correlation
MDA	malondialdehyde
MS	mass spectrometer
MPO	myeloperoxidase
NMR	nuclear magnetic resonance spectrometer
NO	nitric oxide
ROS	reactive oxygen species
SOD	superoxide dismutase

## References

[1] He J.Y., Ma N., Zhu S., Komatsu K., Li Z.Y., Fu W.M. (2015). The genus Codonopsis (Campanulaceae): A review of phytochemistry, bioactivity and quality control. J. Nat. Med..

[2] Sun Y.X., Liu J.C. (2008). Structural characterization of a water soluble polysaccharide from the roots of *Codonopsis pilosula* and its immunity activity. Int. J. Biol. Macromol..

[3] Zhang Y.J., Zhang L.X., Yang J.F., Liang Z.Y. (2010). Structure analysis of water-soluble polysaccharide CPPS3 isolated from *Codonopsis pilosula*. Fitoterapia.

[4] Yang C., Gou Y., Chen J., An J., Chen W., Hu F. (2013). Structural characterization and antitumor activity of a pectic polysaccharide from *Codonopsis pilosula*. Carbohydr. Polym..

[5] Zhao X.N., Hu Y.L., Wang D.Y., Liu J.Z., Guo L.W. (2013). The comparison of immune-enhancing activity of sulfated polysaccharidses from Tremella and Condonpsis pilosula. Carbohydr. Polym..

[6] Zou Y.F., Chen X.F., Malterud K.E., Rise F., Barsett H., Inngjerdingen K.T. (2014). Structural features and complement fixing activity of polysaccharidesfrom *Codonopsis pilosula* Nannf. var. modesta L.T.Shen roots. Carbohydr. Polym..

[7] Liu C., Chen J., Li E.T., Fan Q., Wang D.Y., Li P., Li X., Chen X., Qiu S., Gao Z. (2015). The comparison of antioxidative and hepatoprotective activities of *Codonopsis pilosula* polysaccharide (CP) and sulfated CP. Int. Immunopharmacol..

[8] Roberfroid M.B., Delzenne N.M. (1998). Dietary fructans. Annu. Rev. Nutr..

[9] Kelly G. (2008). Inulin-type prebiotics—A review Part 1. Altern. Med. Rev..

[10] Kelly G. (2009). Inulin-type prebiotics—A review Part 2. Altern. Med. Rev..

[11] Vandeputte D., Falony G., Vieira-Silva S., Wang J., Sailer M., Theis S., Verbeke K., Raes J. (2017). Prebiotic inulin-type fructans induce specific changes in the human gut microbiota. Gut.

[12] Caleffi E.R., Krausova G., Hyrlova I., Paredes L.L.R., Santos M.M., Guilherme Lanzi Sassaki G.L., Gonçalves R.A.C., de Oliveira A.J.B. (2015). Isolation and prebiotic activity of inulin-type fructan extracted from Pfaffia glomerata (Spreng) Pedersen roots. Int. J. Biol. Macromol..

[13] Guth P.H., Aures D., Paulsen G. (1979). Topical aspirin HCl gastric lesions in the rat, cytoprotective effect of prostaglandin, cimetidine, and probanthine. Gastroenterology.

[14] Xu J., Chen D., Liu C., Wu X.Z., Dong C.X., Zhou J. (2016). Structural characterization and anti-tumor effects of an inulin-typefructan from Atractylodes chinensis. Int. J. Biol. Macromol..

[15] Cerantola S., Kervarec N., Pichon R., Magne C., Bessieresa M.A., Deslandes E. (2004). NMR characterisation of inulin-type fructooligosaccharides as the major water-soluble carbohydrates from *Matricaria maritima* (L.). Carbohydr. Res..

[16] Lacy E.R., Ito S. (1982). Microscopic analysis of ethanol damage to rat gastric mucosa after treatment with a prostaglandin. Gastroenterology.

[17] Stewart D.J., Ackroyd R. (2011). Peptic ulcers and their complications. Surgery.

[18] Arab H.H., Salama S.A., Omar H.A., Arafa E.S.A., Maghrabi I.A. (2015). Diosmin Protects against Ethanol-Induced Gastric Injury in Rats: Novel Anti-Ulcer Actions. PLoS ONE.

[19] Li W., Wang X., Zhang H., He Z., Zhi W., Liu F., Wang Y., Niu X. (2016). Anti-ulcerogenic effect of cavidine against ethanol-induced acute gastric ulcer in mice and possible underlying mechanism. Int. Immunopharmacol..

[20] Ibrahim I.A., Abdulla M.A., Hajrezaie M., Bader A., Shahzad N., Al-Ghamdi S.S., Gushash A., Hasanpourghadi M. (2015). The gastroprotective effects of hydroalcoholic extract of Monolluma quadrangula against ethanol-induced gastric mucosal injuries in Sprague Dawley rats. Drug Des. Dev. Ther..

[21] Medeiros J.V., Gadelha G.G., Lima S.J., Garcia J.A., Soares P.M., Santos A.A., Brito G.A.C., Ribeiro R.A., Souza M.H.L.P. (2008). Role of the NO/cGMP/KATP pathway in the protective effects of sildenafil against ethanol-induced gastric damage in rats. Br. J. Pharmacol..

[22] Avery S.V. (2011). Molecular targets of oxidative stress. Biochem. J..

[23] Lykkesfeldt J. (2007). Malondialdehyde as biomarker of oxidative damage to lipids caused by smoking. Clin. Chim. Acta.

[24] Cadirci E., Suleyman H., Aksoy H., Halici Z., Ozgen U., Koc A., Ozturk N. (2007). Effects of Onosma armeniacum root extract on ethanol-induced oxidative stress in stomach tissue of rats. Chem. Biol. Interact..

[25] Martin M.J., Jimenez M.D., Motilva V. (2001). New issues about nitric oxide and its effects on the gastrointestinal tract. Curr. Pharm. Des..

[26] Dursun H., Bilici M., Albayrak F., Ozturk C., Saglam M.B., Alp H.H., Suleyman H. (2009). Antiulcer activity of fluvoxamine in rats and its effect on oxidant and antioxidant parameters in stomach tissue. BMC Gastroenterol..

[27] Suo H.Y., Feng X., Zhu K., Wang C., Zhao X., Kan J.Q. (2015). Shuidouchi (Fermented Soybean) Fermented in Different Vessels Attenuates HCl/Ethanol-Induced Gastric Mucosal Injury. Molecules.

[28] Nishida K., Ohta Y., Ishiguro I. (1998). Contribution of NO synthases to neutrophil infiltration in the gastric mucosal lesions in rats with water immersion restraint stress. FEBS Lett..

[29] Takeuchi K., Yasuhiro T., Asada Y., Sugawa Y. (1998). Role of nitric oxide in pathogenesis of aspirin-induced gastric mucosal damage in rats. Digestion.

